# Supplementary material for: Trend of genital ulcers and discharge and associated factors among survey respondents in Tanzania, 2004–2022: Analysis of demographic health surveys
Source: PLoS One. 2025 Apr 16;20(4):e0319467. doi: 10.1371/journal.pone.0319467 (PMC12002491; doi:10.1371/journal.pone.0319467)
Supplement: Attachment — Submitted filename: Response to reviewers.docx [file pone.0319467.s002.docx]

Dear editors PLOS ONE journal,

This letter serves as response to reviewer’s comments for the manuscript “**Trend of genital ulcers and discharge and associated factors sexually transmitted infections among survey respondents in Tanzania, 2004-2022: Analysis of demographic health surveys.**” We greatly appreciate editors and reviewers and believe their comments have significantly improved our manuscript.

Below are point by point responses

1. Please ensure that your manuscript meets PLOS ONE's style requirements

The manuscript meets PLOS ONE’s style requirements

2. Please include a complete copy of PLOS’ questionnaire
   a. A copy of PLOS questionnaire has been provided
3. Data Availability Statement
   a. DHS data is publicly available at DHS website upon request. We have provided a link to where raw dataset can be requested and downloaded.
4. Please amend your manuscript to include your abstract after the title page
   a. We have included abstract after tittle page.

Reviewer #1: Summary
The authors evaluated trends in the prevalence of STIs using the 2004, 2010 and 2022 Tanzania using Demographic Health Survey (TDHS) data. Participants aged 15 to 49 years who reported having had genital ulcers and/or discharge within 12 months prior to the survey were included in the analysis.

General Comment
The manuscript is clearly written and addresses an important topic.
It could however benefit from some edits.

Title: This analysis only evaluated data for respondents who had genital ulcers and/or discharge, not all STIs in general. Therefore, consider editing the title to read as follows; Trend of genital ulcers and genital discharge respondents in Tanzania: Analysis of demographic health surveys.

**We thank you for your comments. We have made the changes as advised.**

Also consider editing the conclusion to convey that the reported increase in prevalence was for ulcers and/or discharge, not all symptomatic STIs. Symptoms of STIs go beyond these two.

**We thank you for your comments. We have made the changes as advised.**

Variable definition:
Line 104-105: It is stated that a person was considered to ever had STI’s if he/she reported the presence of genital ulcers and/or discharge. Time dimension is missing in this definition. Please edit to improve clarity.

**We thank you for your comments. We have included time frame to improve clarity.**

Discussion:
Ensure that the range of STIs covered in the studies referenced for comparison was the same as for TDHS (i.e. genital ulcers and/or discharge).

**We thank you for your comments. Where we made direct comparisons, all references included were related to the same symptoms used for our study. Moreover, comparison were made using demographic health surveys data from different countries such as Uganda, Kenya, Mali and Malawi.**

Ensure that all recommendations are supported by the findings of the study.

**We thank you for your comments. We have taken note and edit accordingly.**

Reviewer #2: Major considerations
• Why only the three TDHS reports were included as far as other data sets are available? or is there a specific reason for not including other available datasets? It would be helpful to explicitly mention the reason, otherwise all available dataset should be included to have a reliable trend analysis.

**We thank you for your comments. DHS are usually conducted every 5 years. However, the 2015/2016 TDHS publicly available dataset does not contain variables related to abnormal discharge and genital ulcers. We agree having more data would improve trend analysis, however, we are limited with the availability of the variables in the shared datasets. We have added this information in the methods.**

• Factors contributing for trends of STI should be highlighted on the title as well as introduction part for consistency. Identified factors associated with STIs globally and regionally from previous report should be mentioned in the introduction part, too. Better to introduce the importance of understanding not just only the prevalence but also the factors contributing to STIs to inform targeted interventions.

**We thank you for your comments. We have provided details for factors associated with increased STI risk and narrated the importance of further exploration.**

• DHS survey is a hierarchical or nested data (respondents are nested from clusters), suggesting the presence intra-group correlations. Using ordinary regression analysis brings a biased estimate, hence hierarchical analysis models should be utilized, as they account for clustering effect.

**We thank you for your comments. Initial analysis did not use ordinary logistic regression, but regression models used to analyze survey datasets that accounted for survey weights and clustering. We have added hierarchical multivariable models and introduced a new table 4 in the revised manuscript**.

• According to the TDHS 2022 data (as of the 2022 Demographic and Health Survey and Malaria Indicator Survey report), “14% of women and 13% of men aged 15–49 years reported having an STI or symptoms of an STI in the 12 months preceding the survey” However, the author reported a prevalence of STIs of 7.5% in men and 9.9% in women (Table 2). How should this discrepancy be addressed, or is there any justification the authors considered? Similarly, a discrepancy for other TDHS reports.

**We thank you for your comments. TDHS had three questions related to STI. (Had any STI in last 12 months; Had genital sore/ulcer in last 12 months and Had genital discharge in last 12 months). The interest for this analysis was to look for symptomatology and focused on the last two questions. The discrepancy in the reporting is likely due to the exclusion of the first question that generally asked presence of STI without specific symptoms. We made changes throughout the manuscript emphasizing genital ulcers/discharge.**

Minor comments
- [ Title]: Given that the information in study is based on self-reported data and does not reflect confirmed diagnoses of STIs, let modify the title. Also consider including the time frame in the title. (e.g., “Trends in Self-reported Sexually Transmitted Infections among Tanzanian Survey Respondents, 2004-2022: Analysis of Demographic Health Surveys”).

**We thank you for your comments: We made edits to the title considering comments from both reviewers.**

- [Abstract]- Using abbreviation is not recommended.

**We thank you for your comments. Due to the word limit, we are inclined to include a few common abbreviations. We have deleted uncommon abbreviations from the abstract.**

- [ Introduction]: the introduction could benefit from a more structured flow. Consider separating the global context from the regional context to improve readability.

**We thank you for your comments. We have made two separate paragraphs. First providing global context, then regional context as advised.**

- [ Result]: Factors reported in the TDHS such as circumcision, women’s justification for using a condom, awareness of STIs, substance use, and others that may potentially be associated with STIs, should also be considered in the regression analysis. Including these variables could provide a more comprehensive understanding of the factors influencing STI prevalence and help identify additional significant predictors.

**We thank you for your comments, we have added a few more variables as advised.**

- [ Discussion]:
o It would be beneficial to provide more context on why the increased trend might have occurred, especially between 2010 and 2022. Are there known changes in behavior, policy, or healthcare access during this period that could explain the trends?

**We thank you for your comments. These are critical questions; however, we may not be able to explain with certainty the cause of increments of these symptomatic STI. We have added a description of these findings.**

o The discussion could also benefit from a comparison with findings from other countries or regions. How do the trends in Tanzania compare with other parts of sub-Saharan Africa or globally? This would help position the findings within a broader context.

**We thank you for your comments. We have reviewed DHS reports of other countries and provided prevalences of symptomatic STIs.**

o A significant association of STIs with some predictor variables has been reported. The author would benefit from explaining the implications and justifications for these associations.

**We thank you for your comments. We have provided implications for some variables. For example, integration of HIV and STI services to identify new infections.**

- [Conclusion]: could be more action-oriented, suggest specific interventions that could help address the rising trend of STIs.

**We thank you for your comments. We have made edits at this section.**

Line [71] “Moreover, STIs increases risk of cancer (HPV and cervical/anal/head and neck cancer) (5,6)”. How STIs increase the risk of head and neck cancers? evidence? even the provided references not support the statement.

**We thank you for your comments. This was in reference to Human papilloma viruses which are known to cause several cancers including head and neck. We have deleted this information to minimize confusion.**

Line 77-78: “Prospectively collected data from eight health care facilities in Tanzania”……What about the consecutive TDHZ reports?

**We thank you for your comments. We have added data for two consecutive surveys at this section.**

Line 101-102: How participants who were unsure about their STI status were handled beyond just exclusion?

**We thank you for your comments. 166 participants who were unsure of having symptoms of STI accounted for 0.4% of the total sample size. We do not anticipate their inclusion would have significant impact. Furthermore, exploration of their demographic characteristics showed similar distributions to those included. For example, median age 26 vs 27 years, rural residence 67% vs 68% among those excluded vs those included in the analysis.**

Line 105-106: The rational for categorization of some variables (e.g. age) could be better justified and cited.

**We thank you for your comments. We justified categorization of some variables and made citations where appropriate**

Line 120-122: Criteria to included variables for the final multivariable model is not clear. If the number of variables is large enough, better to use a statistical criterion (often using a p-value threshold, p < 0.25 or < 0.20) to reduce the number of variables considered, otherwise all variables should be included in the final multivariable regression analysis.

**We thank you for your comments. We have made changes in this section.**

- Including model fitness tests and model assumption tests in the statistical analysis section is crucial for ensuring the validity of the result.

**We thank you for your comments, we have included how we assessed fitness of the models in the analysis section.**
