# Supplementary material for: Trend of genital ulcers and discharge and associated factors among survey respondents in Tanzania, 2004–2022: Analysis of demographic health surveys
Source: PLoS One. 2025 Apr 16;20(4):e0319467. doi: 10.1371/journal.pone.0319467 (PMC12002491; doi:10.1371/journal.pone.0319467)
Supplement: Attachment — Submitted filename: Response_to_reviewers_auresp_2.docx [file pone.0319467.s003.docx]

Dear editors PLOS ONE journal,

This letter serves as response to reviewer’s comments for the manuscript “**Trend of genital ulcers and discharge and associated factors among survey respondents in Tanzania, 2004-2022: Analysis of demographic health surveys.**” We greatly appreciate editors and reviewers and believe their comments have significantly improved our manuscript.

Below are point by point responses

Reviewer #1: The authors have addressed my comments satisfactorily in their response. I also noted satisfactory response to crosscutting comments raised by the second reviewer and me.

**We are grateful that we responded your earlier comments.**

Reviewer #2: Major concern,
My major concern regarding the statistical analysis remains unresolved. The hierarchical models as the authors present in the revised manuscript (Model 1: Survey year, Model 2: Demographic characteristics + Survey year, Model 3: Risk behavior + other characteristics) lack clarity and do not align with the appropriate methodology for context of the study. For a better analysis in this type of setting, they should consider appropriate hierarchical models like multilevel regression analysis to account for the hierarchical nature of the data(i.e it accounts for both individual and community-level variations). Its assumption should be checked and if model assumption is satisfied, the following steps are recommended
Model 1 (Intercept-only model)- This should be the baseline model to identify whether there is significant variability between communities and individuals in the outcomes of interest (genital ulcers and discharge).
Model 2: Model that includes only individual-level characteristics (e g. age, sex…
Model 3: Model that introduces community-level variables (eg. Like Residence, community education etc. …..).
Model 4: A full model that includes both individual- and community-level characteristics.
The authors should rigorously check the assumptions of multilevel regression and use appropriate model selection criteria like AIC/BIC/PCV to determine the best-fitting model. In doing so, it could assess how much variance is explained at different levels and select the best-fitting model for final interpretation. Without proper justification and methodological rigor, the current analysis is insufficient to have accurate and interpretable results regarding the associated factors. Hence, a thorough revision of the statistical approach is essential for addressing this major concern.

**We thank you for your comments. We needed clarification on which variables were considered community vs individual characteristics. Based on the information provided. It is only survey year that is categorized as community level variable. For this analysis we used the terms level-1 and level-2 variables for individual characteristics and survey year respectively. The dataset was analyzed as suggested and conducted three hierarchical models as opposed to four since having a model with survey year alone would not make these models hierarchical.**
