# Supplementary material for: Trend of genital ulcers and discharge and associated factors among survey respondents in Tanzania, 2004–2022: Analysis of demographic health surveys
Source: PLoS One. 2025 Apr 16;20(4):e0319467. doi: 10.1371/journal.pone.0319467 (PMC12002491; doi:10.1371/journal.pone.0319467)
Supplement: Attachment — Submitted filename: Response_to_reviewers_auresp_3.docx [file pone.0319467.s004.docx]

Dear editors PLOS ONE journal,

This letter serves as response to reviewer’s comments for the manuscript “**Trend of genital ulcers and discharge and associated factors among survey respondents in Tanzania, 2004-2022: Analysis of demographic health surveys.**” We greatly appreciate editors and reviewers and believe their comments have significantly improved our manuscript.

Below are point by point responses

Abstract
• Line 42- its better to refer to factors associated with genital ulcers/discharge in the past 12m as opposed to STIs. I think one of the reviewers recommended a change in title but I think it should go beyond the title.
• Line 45- refer to prevalence of ulcers/discharge

***We thank you for your comments, we have taken note and made changes throughout the manuscript.***

Introduction
• Line 78- state whether the study of STI in the 8 health facilities included people who were symptomatic or asymptomatic.

***We thank you for your comments. Prevalence of the stated infections were from patients who presented with genital symptoms. We have added this information as advised.***

• Line 79- did the 2004 Tanzanian DHS test for STIs?

***We thank you for your comments, the 2004 DHS did not test for STI, but asked survey respondents if they had STI in the last 12 months. We have edited the statement to reflect these were self-reported data.***

• Line 82- add something about how STIs are managed in Tanzania. If its syndromic management, add information about its accuracy in predicting the presence of an STI among people with genital discharge or ulcers.

***We thank you for your comments. We have added this information as advised.***

Methods
• Line 114- might be more accurate to refer to the outcome as prevalence of ulcers/ discharge OR prevalence of STI-related symptoms. This is because the presence of an ulcer or discharge does not equate to an STI particularly for women or those with ulcers.

***We thank you for your comments, we have changed our outcome variable to genital ulcers/discharge throughout the manuscript.***

• Line 132 - the authors dont say what variables were used for weighting.

***We thank you for your comments. Normally DHS uses cluster, household, individual , the variables related to selection probabilities at each stage of sampling as well as response rates at the household and individual level to calculate weights. These are then adjusted for non-response to create the final survey weights. This information has been added.***

Results
• Line 175- prevalence of genital ulcer/discharge should be outcome and not STI prevalence. Please correct the results section

***We thank you for your comments, we have changed the outcome to be genital ulcers/discharge throughout the manuscript.***

• Line 199- 200- Add to the discussion why you found this? Generally STIs are more prevalenct in younger people but are more likely to be asymptomatic in that group. Prevalence of symptoms and not infection increases with age as people acquire immunity

***We thank you for your comments, while this argument is plausible, literature indicates less STI among older individuals is more likely due to behavior and not necessarily immunity.***

• Line 216 - women are more likely to have non-STI causes of ulcers/ discharge compared to males males eg vaginal thrush, bacterial vaginosis, allergies etc. Although women are also more likely to have prevalent STI infections as well, it will not to to the same extent as the likelihood of having ulcers/ discharge- Another systematic review showed steady burden of infections in SSA with the exception of chlamydia. (see Michalow, Julia, Hall, Lauren, Rowley, Jane, Anderson, Rebecca, Hayre, Quinton, Chico, R. Matthew, Edun, Olanrewaju, Knight, Jesse, Kuchukhidze, Salome, Majaya, Evidence, Reed, Domonique, Stevens, Oliver, Walters, Magdalene, Peters, Remco, Cori, Anne, Boily, Marie-Claude and Imai-Eaton, Jeffrey DOI: 10.1101/2024.12.16.24319070).

***We thank you for your comments, we added this observations in the discussion***

Discussion
• Lines 270- 277- increasing prevalence of symptoms does not imply increase in STI infection. This part of the discussion needs to be more nuanced. The two systematic reviews quoted show that the prevalence of specific STIs hasnt changed over time (except for chlamydia) among both symptomatics and aymptomatics. So unless there was testing in the surveys, there is no sufficient evidence of increase in infection but of increasing symptoms

***We thank you for your comments, we have edited this section to reflects symptoms of STI and not necessarily STIs. DHS does not test people except for HIV. Other STI data collected is based on self-reported information on history of STI and associated symptoms.***

• Line 278- but receiving an HIV result was not statistically significant in your adjusted model? Is there testing for STIs in Tanzania? A possible explanatiom for this may be that an HIV negative result is associated with continued high risk behaviour or doesnt change existing high risk behaviours hence the prevalence of STI-associated symptoms.

***We thank you for your comments, Testing for STI in Tanzania is often done in high tier hospitals, majority of people are treated syndromically. Because our association between testing and receiving HIV results on genital ulcers/discharge was based on bivariate analysis, this section in the discussion has been deleted.***
